# Genetic Predisposition and Salt Sensitivity in a Chinese Han Population: The EpiSS Study

**DOI:** 10.1155/2020/3167875

**Published:** 2020-02-17

**Authors:** Kuo Liu, Bo Xi, Zheng Liu, Han Qi, Bin Liu, Jie Zhang, Han Cao, Yuxiang Yan, Min Zhao, Yan He, Ling Zhang

**Affiliations:** ^1^Department of Epidemiology and Health Statistics, School of Public Health, Capital Medical University, Beijing 100069, China; ^2^Beijing Municipal Key Laboratory of Clinical Epidemiology, Beijing, China; ^3^Department of Epidemiology, School of Public Health, Shandong University, Jinan, China; ^4^Department of Food Hygiene and Nutrition, School of Public Health, Shandong University, Jinan, China

## Abstract

**Objectives:**

Genome-wide association studies and candidate gene studies have found many single nucleotide polymorphisms (SNPs) that affect salt sensitivity (SS). We constructed a polygenic risk score (PRS) to estimate the joint effect of these SNPs on SS.

**Methods:**

We recruited 762 Chinese participants into the study. An unweighted PRS was constructed using 42 known genetic risk variants associated with SS or salt sensitivity blood pressure. A modified Sullivan's acute oral saline load and diuresis shrinkage test was used to detect salt sensitivity. Logistic regression was used to estimate the joint effect of the SNPs on SS both overall and after stratification by hypertension.

**Results:**

The mean age of the participants was 57.1 years, and most of them were female (77.4%). The prevalence of SS was 28.7%. Both the continuous PRS and PRS tertiles were significantly associated with the risk of SS and a BP increase of more than 5 mmHg during acute salt loading but were not associated with a BP decrease of more than 10 mmHg during the diuresis shrinkage process. In the normotensive group, participants with PRSs in the middle and top tertiles had a more than twofold increased risk of SS (OR = 2.18, 95% CI: 1.15–4.12, *P* = 0.016, and OR = 2.28, 95% CI: 1.19–4.38, *P* = 0.016, and OR = 2.28, 95% CI: 1.19–4.38, *P* = 0.016, and OR = 2.28, 95% CI: 1.19–4.38, *P* = 0.016, and OR = 2.28, 95% CI: 1.19–4.38,

**Conclusion:**

The 42 investigated SNPs were jointly and significantly associated with SS, especially in the normotensive Chinese population. These findings may provide genetic evidence for identifying target populations that would benefit from salt restriction policies.

## 1. Introduction

Salt sensitivity of blood pressure (SSBP) is characterized as individual heterogeneity of the blood pressure (BP) response to salt intake. Abundant evidence has indicated a causal relationship between salt intake and the risks of hypertension and cardiovascular complications [1, 2].

Previous studies have identified various single nucleotide polymorphisms (SNPs) underlying salt sensitivity (SS) [3]. A large family-based and dietary-feeding study found that polymorphisms of the FAM84A and VSNL1 genes were associated with SSBP [4]. Genome-wide association studies (GWAS) and candidate gene studies found evidence of an association between genes of other pathways, including a polymorphism of ENaC subunits in ion and water channels [5], an NAD(P)H oxidase p22phox gene C242T polymorphism in the endothelial system [6], a beta-2 adrenergic receptor diplotype in the sympathetic nervous system [7], and SSBP. In these previous studies, each SNP only contributed a minor effect on the blood pressure response in salt loading/depletion, and the results were inconsistent. We hypothesize that the joint association of several BP genes of different pathways on SS is stronger than that of individual genetic variants. A polygenic risk score (PRS) indicates the likelihood of an outcome developing due to a person's genome and can reflect a general genetic susceptibility of a certain disease [8]. To date, evidence regarding the association between the PRS and salt sensitivity remains limited, and the results are inconsistent (see below).

The GenSalt study [7] investigated the association between a PRS based on multiple BP or SSBP susceptibility SNPs and the BP response in chronic salt loading/depletion. This study found an inverse relationship between the PRS and SSBP. Another validation study of candidate genes showed a positive relationship between the PRS and salt sensitivity hypertension with a relatively small sample size [9]. It is known that the blood pressure response to chronic and acute salt loading involves different mechanisms. Acute (but not chronic) sodium loading may have deleterious microvascular effects by hydrostatic, hypertonic, or direct effects of sodium on the endothelium [10]. The purpose of this study was to estimate the association between a multilocus PRS and salt sensitivity. Knowing the genetic variants jointly associated with SS could help to identify populations that would benefit from salt restriction, which could promote the practice of precision medicine. Moreover, incorporating SS genetic risk information into clinical practice may refine risk estimates and aid in the prevention of SS-related diseases, such as hypertension and cardiovascular disease.

## 2. Methods

### 2.1. Study Population and Data Collection

The study was conducted in 7 communities from July to August 2015 in Liao Ning Province. Participant's inclusion criteria were as follows: (1) unrelated Chinese Han residents who had been living in place of residence for more than 5 years; (2) 35–70 years old; (3) early essential hypertension diagnosed by a secondary or tertiary hospital according to the 2010 Chinese Hypertension Guidelines (160 mmHg > SBP ≥ 140 mmHg) and (or) 100 mmHg > DBP ≥ 90 mmHg); and (4) not taking antihypertension medicine for at least 24 hours before attending our survey. The exclusion criteria were as follows: (1) patients with severe hypertension (SBP > 160 mmHg and (or) DBP > 100 mmHg); (2) participants with cardiac insufficiency, cardiomyopathy, valvulopathy, congenital heart disease, myocardial infarction, stroke, type 2 diabetes mellitus, hepatopathy, nephropathy, or cancer; (3) pregnant or breastfeeding women; and (4) participants with a low sodium diet for more than 1 year. Our protocol was approved by the Ethics Committee of Capital Medical University. Written informed consent was obtained from all participants. A total of 762 hypertension patients were recruited.

A questionnaire was used to collect demographic characteristics (birthday, sex, etc.), lifestyle risk factors (cigarette smoking, etc.), medical history of chronic disease, and medication history. Interviewers were trained in a standardized way to perform face-to-face interviews with the questionnaire. Smoking was defined as at least 1 cigarette per day for more than 1 year. Those participants who had quit smoking for at least 3 months were defined as past smoking. Mercury sphygmomanometers were used to measure blood pressure. Participants who had just smoked, exercised, or drank coffee were asked to rest for at least 30 minutes before the measurement. Two technicians were trained in BP measurement following the recommendations of the American Heart Association, and they measured the blood pressure of all the participants in this study. Mean arterial pressure (MAP) was used to represent blood pressure. MAP was calculated according to the standard formula: MAP = (SBP + 2 × DBP)/3. Each patient was asked to collect a 24-hour urine sample. After the first urine at 6 : 00 AM, participants were asked to collect all urine from then on until the next day at 6 : 00 AM. One milliliter of urine was derived from the 24-hour urine to measure the concentration of Na^+^. Hypertensive patients were defined as those with SBP ≥ 140 mmHg or DBP ≥ 90 mmHg at the baseline measurement of the survey or participants who had been diagnosed with hypertension in the hospital and regularly took antihypertensive medicine. Diabetic patients were defined as participants with fasting glucose ≥7.0 mmol/L on the survey or who had been diagnosed with diabetes in the hospital and regularly took hypoglycemic drugs. Dyslipidemia was defined by meeting at least one of the following criteria: total cholesterol concentration more than 5.2 mmol/L, triglyceride concentration more than 1.7 mmol/L, low-density lipoprotein cholesterol concentration more than 3.4 mmol/L, or high-density lipoprotein cholesterol less than 1.0 mmol/L.

### 2.2. Assessment of Salt Sensitivity

A modified Sullivan's method, which has been previously used in a Chinese population, was used to assess salt sensitivity [11, 12]; refer our previously published paper for more details [13]. Fasting participants were asked to orally intake 1000 ml 0.9% NaCl followed by 40 mg furosemide orally within 30 minutes at 8 : 00 AM. Blood pressure was again measured 2 hours later. An MAP increase of more than 5 mmHg after oral salt loading or an MAP reduction of more than 10 mmHg after the diuresis shrinkage process was considered salt sensitivity [14].

### 2.3. SNP Selection

We selected 42 recently identified SS susceptibility loci from published GWAS or candidate gene papers before April 2016. Five susceptibility loci associated with salt-sensitive blood pressure identified in genome-wide linkage scans and GWAS in the Chinese Han population were included [4, 15], and the other 37 loci were from several pathways: 4 SNPs in the RAAS system, 1 SNP in the Kallikrein–Kinin system, 15 SNPs in the ion channel system, 2 SNPs in the apelin system, 7 SNPs in the endothelial system, 3 SNPs in the intracellular messenger system, 2 SNPs in the sympathetic nervous system, and 3 SNPs in undefined pathways (see Supplementary [Supplementary-material supplementary-material-1]).

### 2.4. SNP Genotyping

Fasting venous blood was collected in 5 ml EDTA tubes, and genomic DNA was isolated with a QIAamp DNA Blood Mini Kit (Tiangen Inc., Hilden, Germany). Genotyping was performed with the Sequenom MassARRAY iPLEX platform (Sequenom, Inc. San Diego, CA, USA). A successful genotyping rate of over 90% was achieved for all 42 SNPs. All samples had an OD260/OD280 value between 1.7 and 2.0, which indicates a DNA concentration over 10 ng/*μ*l. All SNPs in this report had a genotyping success rate of >95%. All SNPs in our study were in Hardy–Weinberg equilibrium (HWE) in the controls (*P* > 0.05).

### 2.5. Statistical Analyses

Normally distributed continuous variables are reported as the mean ± standard deviation (SD), and nonnormally distributed continuous variables are reported as the median ± quintiles. Student's *t*-test was used to examine the difference between two groups of continuous variables. The PRS was calculated by summing the number of risk alleles at each polymorphic locus. The PRS was examined both as a continuous variable and as a categorical variable categorized by PRS tertile to ensure the reliability of the results. Participants with missing genotypes (15 controls and 8 cases) were excluded from the polygenic risk score calculation. Unconditional logistic regression was used to estimate the PRS effect size after adjusting for traditional risk factors, including age, sex, smoking, BMI, dyslipidemia, and history of diabetes. All analyses were conducted in IBM SPSS Statistics 19.0 software (SPSS, Chicago, IL, USA). A two-sided *P* value < 0.05 was considered statistically significant.

## 3. Results

### 3.1. Characteristics of the Study Population

Table 1 shows the characteristics of the participants by salt sensitivity status. Current smokers and hypertension patients were more likely to develop salt sensitivity. The proportion of hypertension was 35.4% in salt sensitivity cases and 26.8% in salt resistance people (*P*=0.043). The proportions of subjects who were current smokers were 18.3% and 12.2% in salt sensitivity cases and controls, respectively (*P*=0.015). SBP, DBP, and MAP were significantly higher in the salt-resistant group than in the salt-sensitive group (*P*=0.014, *P*=0.008, and *P*=0.006, respectively).

### 3.2. Genetic Effects on Salt Sensitivity Risk

The joint effect of the 42 SNPs on salt sensitivity was evaluated. We calculated an unweighted PRS by summing the numbers of risk alleles. The PRS was significantly associated with the risk of salt sensitivity, and the association was robust to the adjustment for potential confounders (full adjustment: per-unit OR = 1.083, 95% CI, 1.037–1.132, *P* < 0.001). The second PRS tertile (full adjustment: OR = 1.73, 95% CI, 1.10–2.72, *P*=0.019) and third PRS tertile (OR = 1.96, 95% CI, 1.23–3.11, *P*=0.004; Table 2) were associated with an increased risk of salt sensitivity compared with the first PRS tertile.

After stratification by hypertension, the PRS was significantly associated with salt sensitivity in the normotensive population, and this association was robust to adjustment for potential confounders. Compared with the participants with PRSs in the first tertile, participants with PRSs in the second tertile (OR = 2.18, 95% CI, 1.15–4.12, *P*=0.016) and third tertile (OR = 2.28, 95% CI, 1.19–4.38, *P*=0.013; Table 2) had an increased risk of salt sensitivity in the normotensive population after adjusting for traditional risk factors. However, the association between the PRS and salt sensitivity was not significant in the hypertension group. In the combined sample, both the PRS and PRS tertiles were significantly associated with a BP increase of more than 5 mmHg during acute salt loading but were not associated with a BP reduction of more than 10 mmHg during the diuresis shrinkage process. In the full adjustment model, the PRS was associated with a 9.1% increased risk of a BP increase of more than 5 mmHg during acute salt loading (OR = 1.09, 95% CI, 1.04–1.14, *P* < 0.001; Table 3). The second and third tertiles of PRS increased the risk of a BP increase >5 mmHg during acute salt loading by 75.4% and 101%, respectively (OR = 1.75, *P*=0.018; OR = 2.01, *P*=0.004; Table 3).

In the normotensive group, the PRS was significantly associated with a BP increase of more than 5 mmHg during acute salt loading after adjustment for potential confounders (OR = 1.15, 95% CI, 1.05–1.19, *P*=0.001). In the process of acute salt loading, participants with PRSs in the second tertile (OR = 1.94, 95% CI, 1.01–3.71, *P*=0.046) and third tertile (OR = 2.30, 95% CI, 1.19–4.44, *P*=0.013; Table 3) had an increased risk of salt sensitivity compared with those with PRSs in the first tertile. In the hypertension group, neither the PRS nor the PRS tertile was significantly associated with BP response to salt loading or diuresis shrinkage.

### 3.3. Traditional Risk Factors of Salt Sensitivity

In the combined sample, smoking was significantly associated with salt-sensitive hypertension (OR = 2.24, 95% CI, 1.03–4.88, *P*=0.042). After stratification by hypertension, the association of smoking with SS was attenuated. The PRS was significantly associated with SS only in the normotensive group. The second and third tertiles of PRS increased the risk of SS by 1.18-fold and 1.28-fold, respectively; details are shown in Table 4.

## 4. Discussion

Our study is one of the few to investigate the joint effect of BP loci on salt sensitivity, which could provide evidence of a high-risk population when implementing salt restriction strategies. We examined 42 SNPs from susceptibility loci for SSBP or SSH. We constructed a PRS using the 42 SNPs to evaluate the joint effect of these SNPs on salt sensitivity. The PRS was found to be significantly associated with SS for the general population and normotensive people but not for hypertensive patients. For the general population and normotensive people, the PRS also showed a significant association with MAP increases of more than 5 mmHg in the process of acute salt loading. The results were robust to the PRS being analyzed as a continuous variable or categorical tertiles.

Our study demonstrated that the effect of the PRS on BP response varies between hypertension and normotensive people. Our findings were consistent with those reported by Weinberger and Fineberg [16], who demonstrated that baseline blood pressure could impact the change in MAP after sodium and volume depletion. The GenSalt group also found that elevated baseline BP levels increased BP responses to dietary sodium intervention [17]. Given that high BP can affect BP responses to sodium loading and that most of the SNPs selected in the current study are associated with the physiological mechanism of hypertension, it is possible that hypertension is a potential confounder in examining the relationship between the PRS and SS. We therefore stratified our population into hypertension and normotensive groups to investigate the effect of the PRS on SS. The association between the PRS and salt sensitivity varied between the hypertension and normotensive populations. The PRS was associated with salt sensitivity in the normotensive population but not in the hypertensive population. Two studies that investigated the joint genetic effect on salt sensitivity had different results from ours. Liu et al. [13] found five SNPs associated with salt sensitivity hypertension, and the five SNPs jointly increased the risk of SS for the hypertension population. However, this study did not use external data to replicate the findings. Evaluating the effect of the PRS on SS in the initial discovery population may have inflated the effect size and led to false-positive results. The results of the GenSalt study [7] were also inconsistent with ours. The GenSalt study found an inverse relationship between the PRS and SSBP. An increased PRS quartile conferred a lower SBP to both the low-sodium and high-sodium intervention groups, which could not be fully explained. There are several differences between the current study and the GenSalt study, which might explain the discordant results. First, the GenSalt study was a family study, including probands and their siblings/spouses/offspring. The current study was a population-based study of unrelated participants. Family studies include participants with not only similar environmental risk factors but also similar genetic backgrounds, which may cause different results from population-based studies. Second, the participants in the GenSalt study underwent a chronic salt loading/salt depletion (7-day low-sodium and 7-day high sodium) intervention. In contrast, the participants in the current study underwent acute sodium loading/salt depletion (oral intake of 1000 ml of 0.9% NaCl within 30 minutes and then oral intake furosemide). In the acute sodium loading process, sodium balance was mainly regulated by the distal nephron, with little contribution from the proximal tubule. In contrast, changes in sodium reabsorption have been observed in both the proximal and distal nephrons in the chronic salt loading process [18]. Because different renal mechanisms of sodium balance are involved in the acute and chronic salt loading/salt depletion processes, the blood pressure response and its genetic determinants could also differ between the acute and chronic protocols [19].

In our study, an increased PRS was associated with greater BP changes during the sodium loading process but was not associated with BP changes in diuresis shrinkage. High salt intake activates the renin-angiotensin-aldosterone system (RAAS). It has been demonstrated that the response of angiotensin II receptor type 1 (AGTR1) gene expression may be relevant for the organism to be able to adapt to salt intake [20]. Abnormal renal tubular reclamation of Na^+^ and Cl^−^ contributes to the anomalous BP change during both salt loading and diuresis shrinkage. The impairment of the Na^+^ excretory ability of the kidney reduces the antihypertensive effect of furosemide and the development of salt-sensitive hypertension [21]. WNK lysine-deficient protein kinase 1 (WNK1), an important modulator of salt homeostasis, regulates the balance between renal Na^+^ reabsorption and K^+^ excretion [22]. The different pathophysiological mechanisms in salt loading/depletion may imply different underlying genetic mechanisms. SNPs in RAAS pathways are related to BP response in salt loading, and other SS-related SNPs are mostly associated with the BP response both in the salt loading and diuresis shrinkage processes. Thus, almost all identified SS-related SNPs have been associated with an abnormal BP response in the salt loading process, but few of them have been related to the BP response in the process of diuresis shrinkage. The underlying genetic mechanisms of BP changes due to diuresis shrinkage need to be further investigated.

In our study, 6 SNPs were nominally associated with SS. SNP rs3754777 in STK39 was associated with salt sensitivity in our study. The serine/threonine kinase 39 gene (STK39) was first identified as a hypertension-susceptibility gene by GWAS [23]. Tang et al. [24] replicated the association of rs3754777 of STK39 with essential hypertension in a male Chinese Han population. Yang et al. [25] conducted a meta-analysis in 2016 and found that smoking was a significant modifier of the association between rs3754777 and hypertension (*P*=0.017). rs3754777 may increase STK39 expression and consequently alter renal Na(+) excretion [20]. SNP rs2638360 in AGTR1 was significantly associated with salt sensitivity in our study. A study found that rs2638360 was associated with essential hypertension in 692 Chinese Hani and 615 Yi minorities [26]. SNP rs12828016 in WNK1 was also found to be associated with salt sensitivity in our study, and our result is consistent with those from a prior Japanese study [22]. In a family study [27], this SNP was found to be significantly associated with the MAP response to a high-sodium intervention (*P*=0.044) and marginally associated with the MAP response to a low-sodium intervention (*P*=0.052).

One of our study's strengths was that 762 hypertensive individuals were recruited to investigate their salt sensitivity. To our knowledge, only the GenSalt study, which aims to identify genetic determinants of salt sensitivity blood pressure, has a larger sample size than ours. However, the GenSalt study focused on genes affecting chronic salt loading, while our study aimed to identify genes associated with BP changes during acute oral saline loading and diuresis shrinkage. The second strength is that the patients in our study were recruited from the community, which may have avoided selection bias to a certain extent. Another strength is that the SNPs included in our study were those previously reported to be associated with salt sensitivity or SSH in the Chinese Han population; thus, the polygenic risk score reflected the genetic background of the Chinese population. Our study also had some limitations. First, we only included genes located on autosomal chromosomes. Some genes, such as the ACE2 gene and AGTR1 gene, both located on the X chromosome, may also contribute to the risk of salt sensitivity. The PRS could not be constructed by simply adding the number of risk alleles if the SNPs were located on the X chromosome because the effects of those SNPs may be different between females and males. A more comprehensive evaluation of genetic risk factors should be conducted in the future. The second limitation is that we did not calculate a weighted PRS. This is because most of the original studies did not report the effect sizes of the SNP effects; therefore, no evident weights were available.

In conclusion, a PRS of 42 SNPs was significantly associated with salt sensitivity and with an increased risk of BP response to acute sodium loading.

## Figures and Tables

**Table 1:** Baseline characteristics between salt-sensitive and salt-resistant groups.

	Salt sensitive (*N* = 219)	Salt resistant (*N* = 543)	*P*
Age (y)	57.16 ± 7.29	57.14 ± 7.41	0.970
Male	58 (26.5)	114 (21.0)	0.104
Smoking			0.015^*∗*^
Current	40 (18.3)	66 (12.2)	
Seldom/never	168 (76.7)	463 (85.3)	
Former smoker	11 (5.0)	14 (2.6)	
SBP (mmHg)	114.22 ± 18.65	117.91 ± 18.61	0.014^*∗*^
DBP (mmHg)	73.89 ± 10.09	76.19 ± 11.11	0.008^*∗*^
MAP (mmHg)	87.33 ± 11.94	90.10 ± 12.72	0.006^*∗*^
Fasting glucose (mmol/L)	5.55 ± 1.13	5.43 ± 0.99	0.135
BMI (kg/m^2^)	25.62 ± 3.29	25.23 ± 3.22	0.135
TC (mmol/L)	5.29 ± 0.98	5.41 ± 0.88	0.126
TG (mmol/L)	1.93 ± 1.14	2.10 ± 1.65	0.150
HDLC (mmol/L)	1.21 ± 0.27	1.23 ± 0.32	0.187
LDLC (mmol/L)	2.68 ± 0.72	2.69 ± 0.66	0.842
Hypertension	57 (35.4)	115 (26.8)	0.043^*∗*^
Diabetes	54 (24.7)	107 (19.7)	0.130
Dyslipidemia	159 (72.6)	423 (77.9)	0.119

SBP, systolic blood pressure; DBP, diastolic blood pressure; MAP, mean arterial pressure; BMI, Body Mass Index; TC, total cholesterol; TG, triglyceride; HDLC, high-density lipoprotein cholesterol; LDLC, low-density lipoprotein cholesterol. ^*∗*^*P* < 0.05.

**Table 2:** Association of the polygenic risk score with salt sensitivity.

	Model A		Model B		Model C	
	OR (95% CI)	*P*	OR (95% CI)	*P*	OR (95% CI)	*P*
*All*						
PRS	1.081 (1.035, 1.129)	<0.001^*∗∗*^	1.082 (1.036, 1.130)	<0.001^*∗∗*^	1.083 (1.037, 1.132)	<0.001^*∗∗*^
PRS tertiles						
1st vs 2nd	1.694 (1.083, 2.652)	0.021^*∗*^	1.702 (1.085, 2.668)	0.020^*∗*^	1.725 (1.095, 2.716)	0.019^*∗*^
1st vs 3rd	1.897 (1.205, 2.985)	0.006^*∗∗*^	1.913 (1.214, 3.015)	0.005^*∗∗*^	1.960 (1.233, 3.113)	0.004^*∗∗*^
*Hypertension*						
PRS	1.052 (0.987, 1.121)	0.117	1.054 (0.989, 1.124)	0.104	1.058 (0.991, 1.129)	0.091
PRS tertiles						
1st vs 2nd	1.377 (0.714, 2.658)	0.340	1.370 (0.708, 2.651)	0.350	1.369 (0.703, 2.666)	0.356
1st vs 3rd	1.582 (0.818, 3.057)	0.173	1.614 (0.832, 3.128)	0.157	1.654 (0.840, 3.260)	0.146
*Normotensives*						
PRS	1.106 (1.042, 1.174)	0.001^*∗∗*^	1.112 (1.048, 1.181)	0.001^*∗∗*^	1.109 (1.044, 1.179)	0.001^*∗∗*^
PRS tertiles						
1st vs 2nd	2.096 (1.123, 3.912)	0.020^*∗*^	2.186 (1.162, 4.114)	0.015^*∗*^	2.179 (1.154, 4.116)	0.016^*∗*^
1st vs 3rd	2.300 (1.219, 4.340)	0.010^*∗*^	2.378 (1.251, 4.522)	0.008^*∗∗*^	2.281 (1.189, 4.376)	0.013^*∗*^

PRS, polygenic risk score. Model A: no variables were adjusted; model B: age and sex adjusted; model C: age, sex, smoking, BMI, dyslipidemia, and DM adjusted. ^*∗*^*P* < 0.05; ^*∗∗*^*P* < 0.01.

**Table 3:** Association of the polygenic risk score with salt sensitivity during the process of acute salt loading and diuresis shrinkage.

	Model A		Model B		Model C	
	OR (95% CI)	*P*	OR (95% CI)	*P*	OR (95% CI)	*P*
*All*						
Acute salt loading^§^						
PRS	1.087 (1.040, 1.137)	<0.001^*∗∗*^	1.089 (1.041, 1.138)	<0.001∗∗	1.091 (1.043, 1.141)	<0.001^*∗∗*^
PRS tertiles						
1st vs 2nd	1.728 (1.090, 2.738)	0.020^*∗*^	1.721 (1.085, 2.732)	0.021^*∗*^	1.754 (1.100, 2.797)	0.018^*∗*^
1st vs 3rd	1.924 (1.207, 3.065)	0.006^*∗∗*^	1.939 (1.215, 3.092)	0.005^*∗∗*^	2.011 (1.251, 3.234)	0.004^*∗∗*^
Diuresis shrinkage^¤^						
PRS	1.058 (0.975, 1.148)	0.175	1.057 (0.974, 1.147)	0.181	1.054 (0.970, 1.144)	0.216
PRS tertiles						
1st vs 2nd	1.481 (0.610, 3.596)	0.386	1.500 (0.617, 3.648)	0.371	1.459 (0.597, 3.568)	0.407
1st vs 3rd	1.781 (0.743, 4.271)	0.196	1.777 (0.740, 4.262)	0.198	1.676 (0.690, 4.069)	0.254
*Hypertension*						
Acute salt loading						
PRS	1.058 (0.991, 1.130)	0.089	1.062 (0.994, 1.134)	0.076	1.062 (0.994, 1.135)	0.076
PRS tertiles						
1st vs 2nd	1.630 (0.832, 3.190)	0.154	1.620 (0.824, 3.187)	0.162	1.608 (0.813, 3.183)	0.172
1st vs 3rd	1.558 (0.786, 3.087)	0.204	1.603 (0.805, 3.193)	0.179	1.623 (0.803, 3.283)	0.178
Diuresis shrinkage						
PRS	0.980 (0.867, 1.107)	0.740	0.981 (0.868, 1.108)	0.756	0.982 (0.865, 1.113)	0.771
PRS tertile						
1st vs 2nd	0.829 (0.226, 3.046)	0.777	0.823 (0.224, 3.029)	0.769	0.819 (0.219, 3.062)	0.766
1st vs 3rd	1.111 (0.326, 3.791)	0.866	1.116 (0.326, 3.818)	0.861	1.190 (0.335, 4.232)	0.788
*Normotensives*						
Acute salt loading						
PRS	1.113 (1.047, 1.184)	0.001^*∗*^	1.117 (1.051, 1.188)	<0.001^*∗∗*^	1.115 (1.048, 1.187)	0.001^*∗∗*^
PRS tertiles						
1st vs 2nd	1.887 (0.995, 3.580)	0.052	1.934 (1.014, 3.685)	0.045^*∗*^	1.939 (1.012, 3.712)	0.046^*∗*^
1st vs 3rd	2.341 (1.229, 4.460)	0.010^*∗*^	2.390 (1.249, 4.572)	0.009^*∗∗*^	2.298 (1.189, 4.442)	0.013^*∗*^
Diuresis shrinkage						
PRS	1.129 (1.007, 1.266)	0.038^*∗*^	1.133 (1.009, 1.272)	0.035^*∗*^	1.135 (1.010, 1.275)	0.034^*∗*^
PRS tertiles						
1st vs 2nd	2.717 (0.702, 10.515)	0.148	2.878 (0.739, 11.211)	0.128	2.988 (0.749, 11.915)	0.121
1st vs 3rd	3.092 (0.797, 11.991)	0.103	3.178 (0.816, 12.380)	0.096	3.025 (0.803, 12.786)	0.099

PRS, polygenic risk score. Model A: no variables were adjusted; model B: age and sex adjusted; model C: age, sex, smoking, BMI, dyslipidemia, and DM adjusted.^§^Logistic regression was used; assignment of dependent variables: MAP raises more than 5 mmHg in the process of acute salt loading assigned to 1, and MAP raises less than 5 mmHg in the process of acute salt loading assigned to 0. Logistic regression was used; assignment of dependent variables: MAP reduces more than 10 mmHg in the process of diuresis shrinkage assigned to 1, and MAP reduces less than 10 mmHg in the process of diuresis shrinkage assigned to 0. ^*∗*^*P* < 0.05;^*∗∗*^*P* < 0.01.

**Table 4:** Effect size of risk factors associated with salt sensitivity.

	SSH vs non-SSH		SSH vs SRH		SSN vs SRN	
	OR (95% CI)	*P*	OR (95% CI)	*P*	OR (95% CI)	*P*
Age (years)	1.019 (0.981, 1.058)	0.338	0.985 (0.949, 1.023)	0.442	0.986 (0.953, 1.020)	0.421
Sex (male)	1.453 (0.681, 3.101)	0.334	1.654 (0.840, 3.260)	0.146	0.505 (0.245, 1.043)	0.065
Smoking habits	2.243 (1.031, 4.880)	0.042^*∗*^	1.795 (0.797, 4.041)	0.158	1.348 (0.595, 3.054)	0.475
Dyslipidemia	1.427 (0.808, 2.521)	0.221	1.057 (0.599, 1.867)	0.848	1.057 (0.599, 1.867)	0.848
Diabetes	1.055 (0.552, 2.016)	0.870	0.789 (0.401, 1.553)	0.493	0.852 (0.487, 1.489)	0.574
BMI	1.072 (0.987, 1.165)	0.100	0.988 (0.908, 1.074)	0.775	1.020 (0.935, 1.112)	0.658
PRS, 1st tertile	Ref	—	Ref	—	Ref	—
PRS, 2nd tertile	0.954 (0.486, 1.872)	0.891	1.369 (0.703, 2.666)	0.356	2.179 (1.154, 4.116)	0.016^*∗*^
PRS, 3rd tertile	1.277 (0.657, 2.482)	0.470	1.654 (1.221, 6.314)	0.146	2.281 (1.189, 4.376)	0.013^*∗*^

SSH, salt sensitivity hypertension; SRH, salt resistance hypertension; SSN, salt sensitivity normotensives; SRN, salt resistance normotensives; BMI, Body Mass Index. Logistic regression was used. Independent variables were age, sex, smoking habits, diabetes, dyslipidemia, BMI, and PRS tertile. ^*∗*^*P* < 0.05.

## Data Availability

In the current research, our dataset includes environmental and genotype data, and all these data were restored in SPSS software. The SPSS data used to support the findings of this study may be requested from the corresponding author, however as the dataset of our research contains genetic information, it requires approval National Human Genetic Resources Administration Office before sharing.

## Supplementary Materials

Supplementary Materials There is one supplementary material file along with our manuscript. There are two tables in this supplementary file. Supplementary Table 1 (or Table S1) provides the details of the candidate genes we selected in the current study, including the pathways of SNPs and the minor/major allele of the SNPs, along with the minor allele frequency. In addition, this table also included the references which had previously reported the association between the SNPs and salt sensitivity blood pressure. In the method section of manuscript, we described the detail of Table S1 in the part of “SNP selection”. Supplementary Table 2 (or Table S2) provides the results of the association of each SNPs and salt sensitivity. In the second part of result section, we briefly described that “Among the 42 SNPs, rs3754777 in STK39,…, and rs16983422 in VSNL1 were nominally associated with salt sensitivity (see supplementary Table S2)”. In Table S2, we proposed our results in detail. The association analysis between each SNPs and SS was conducted by comparing the proportion of different genotypes in salt sensitivity or salt-resistant group, and the association analysis was done when assuming different genetic models. Chi-square test and logistic regression were both used to do association analysis.
