# Supplementary material for: Genetic Predisposition and Salt Sensitivity in a Chinese Han Population: The EpiSS Study
Source: Int J Hypertens. 2020 Feb 17;2020:3167875. doi: 10.1155/2020/3167875 (PMC7048915; doi:10.1155/2020/3167875)
Supplement: Supplementary Materials — There is one supplementary material file along with our manuscript. There are two tables in this supplementary file. Supplementary Table 1 (or Table S1) provides the details of the candidate genes we selected in the current study, including the pathways of SNPs and the minor/major allele of the SNPs, along with the minor allele frequency. In addition, this table also included the references which had previously reported the association between the SNPs and salt sensitivity blood pressure. In the method section of manuscript, we described the detail of Table S1 in the part of “SNP selection”. Supplementary Table 2 (or Table S2) provides the results of the association of each SNPs and salt sensitivity. In the second part of result section, we briefly described that “Among the 42 SNPs, rs3754777 in STK39,…, and rs16983422 in VSNL1 were nominally associated with salt sensitivity (see supplementary Table S2)”. In Table S2, we proposed our results in detail. The association analysis between each SNPs and SS was conducted by comparing the proportion of different genotypes in salt sensitivity or salt-resistant group, and the association analysis was done when assuming different genetic models. Chi-square test and logistic regression were both used to do association analysis. [file 3167875.f1.docx]

Table S1 Characteristics of SNPs included in the study

| Pathway | SNPs | Genes | Reference | Chromosome | Position (bp) | Alleles  (Minor/Major) | MAF | HWE_*P* |
| --- | --- | --- | --- | --- | --- | --- | --- | --- |
| RAAS system | rs699 | *AGT* | [1, 2] | 1 | 230710048 | A/G | 0.201 | 0.168 |
|  | rs1799998 | *CYP11B2* | [3-5] | 8 | 142918184 | G/A | 0.307 | 0.574 |
|  | rs2638360 | *AGTR1* | [5, 6] | 3 | 148710569 | G/A | 0.108 | 0.296 |
|  | rs3772616 | *AGTR1* | [5, 6] | 3 | 148720404 | T/C | 0.158 | 0.71 |
|  |  |  |  |  |  |  |  |  |
| Ion channel system | rs11893826 | *SLC8A1* | [8, 9] | 2 | 40337507 | A/G | 0.296 | 0.922 |
|  | rs434082 | *SLC8A1* | [8-10] | 2 | 40257934 | T/C | 0.136 | 0.66 |
|  | rs6112470 | *SLC24A3* | [8, 9] | 20 | 19581935 | C/T | 0.213 | 0.244 |
|  | rs2286007 | *WNK1* | [11, 12] | 12 | 862125 | T/C | 0.079 | 0.122 |
|  | rs12828016 | *WNK1* | [11, 12] | 12 | 889199 | T/G | 0.256 | 0.445 |
|  | rs2255390 | *WNK1* | [11, 12] | 12 | 890596 | G/A | 0.44 | 0.898 |
|  | rs880054 | *WNK1* | [11, 12] | 12 | 879392 | C/T | 0.261 | 0.119 |
|  | rs2288774 | *NEDD4L* | [11, 13] | 18 | 58316098 | C/T | 0.358 | 0.444 |
|  | rs1010069 | *CLCNKA* | [14] | 1 | 16026442 | G/A | 0.283 | 0.971 |
|  | rs1805152 | *CLCNKA* | [14] | 1 | 16030006 | G/A | 0.284 | 0.859 |
|  | rs848307 | *CLCNKA* | [14] | 1 | 15992737 | A/G | 0.264 | 0.626 |
|  | rs35929607 | *STK39* | [15, 16] | 2 | 168179226 | A/G | 0.42 | 0.787 |
|  | rs3754777 | *STK39* | [15, 16] | 2 | 168159404 | T/C | 0.255 | 0.567 |
|  | rs1937506 | *STK39* | [15, 16] | 13 | 67461239 | A/G | 0.123 | 0.481 |
|  | rs6749447 | *STK39* | [15, 16] | 2 | 168184876 | T/G | 0.318 | 0.125 |
| Kallikrein–kinin system | rs5516 | *KLK1* | [7] | 19 | 50820217 | C/G | 0.213 | 0.912 |
| Apelin system | rs721608 | *LOC105369309* | [17] | 11 | 57230449 | G/A | 0.133 | 0.574 |
|  | rs746886 | *APLNR* | [17] | 11 | 57234637 | A/G | 0.396 | 0.603 |
| Endothelial system | rs1799983 | *NOS3* | [18] | 7 | 150999023 | T/G | 0.104 | 0.325 |
|  | rs5351 | *EDNRB-AS1* | [19] | 13 | 77901178 | C/T | 0.395 | 0.711 |
|  | rs11161637 | *DDAH1* | [20] | 1 | 85555486 | G/A | 0.248 | 0.179 |
|  | rs4673 | *CYBA* | [21, 22] | 16 | 88646828 | A/G | 0.077 | 0.834 |
|  | rs9371562 | *ESR1* | [23] | 6 | 151939507 | A/T | 0.094 | 0.71 |
|  | rs9383951 | *ESR1* | [23] | 6 | 151974478 | C/G | 0.094 | 0.691 |
|  | rs2681472 | *ATP2B1* | [16] | 12 | 89615182 | G/A | 0.367 | 0.546 |
| Intracellular messenger system | rs1129649 | *GNB3* | [24] | 12 | 6839304 | C/T | 0.302 | 0.783 |
|  | rs1904694 | *PRKG1* | [25] | 10 | 51145734 | G/A | 0.329 | 0.774 |
|  | rs7897633 | *PRKG1* | [25] | 10 | 51197961 | A/G | 0.43 | 0.211 |
| Sympathetic nervous system | rs1042713 | *ADRB2* | [7, 26, 27] | 5 | 148826877 | A/G | 0.438 | 0.053 |
|  | rs1024323 | *GRK4* | [1, 28] | 4 | 3004316 | T/C | 0.175 | 0.806 |
| GWAS identified | rs16998073 | *FGF5* | [29] | 4 | 80263187 | T/A | 0.371 | 0.3 |
|  | rs16983422 | *VSNL1* | [29] | 2 | 17263396 | G/A | 0.13 | 0.123 |
|  | rs11674786 | *FAM84A* | [29,30] | 2 | 14360378 | C/A | 0.107 | 0.697 |
|  | rs16890334 | *IRAK1BP1* | [30] | 6 | 78846449 | C/T | 0.073 | 0.258 |
|  | rs7577262 | *TRPM8* | [30] | 2 | 233910224 | A/G | 0.339 | 0.171 |
| Others | rs2758151 | *SGK* | [31, 32] | 6 | 134166530 | T/C | 0.474 | 0.702 |
|  | rs1045642 | *ABCB1* | [33] | 7 | 87509329 | A/G | 0.404 | 0.633 |
|  | rs2398162 | *NR2F2-AS1* | [16] | 15 | 96287321 | A/G | 0.377 | 0.222 |

Abbreviations: SNP, Single nucleotide polymorphisms.

**Reference:**

[1] Lee M, Mi K K, Kim S M, et al. Gender-Based Differences on the Association between Salt-Sensitive Genes and Obesity in Korean Children Aged between 8 and 9 Years[J]. Plos One, 2015, 10(3):e0120111.

[2] Schorr U, Blaschke K, Beige J, et al. Angiotensinogen M235T variant and salt sensitivity in young normotensive Caucasians.[J]. Journal of Hypertension, 1999, 17(4):475-9.

[3] Xu H, Wang X, Liu M, et al. Association of aldosterone synthase (CYP11B2) -344 T/C polymorphism with diabetic nephropathy: A meta-analysis[J]. Journal of the renin-angiotensin-aldosterone system : JRAAS, 2016, 17(1):1470320316633896.

[4] Iwai N, Kajimoto K, Tomoike H, et al. Polymorphism of CYP11B2 determines salt sensitivity in Japanese.[J]. Hypertension, 2007, 49(4):825-31.

[5] Pamies-Andreu E , Ramirez-Lorca R , Stiefel García-Junco, P, et al. Renin-angiotensin-aldosterone system and G-protein beta-3 subunit gene polymorphisms in salt-sensitive essential hypertension[J]. JOURNAL OF HUMAN HYPERTENSION, 2003, 17(3):187-191.

[6] Gu D, Kelly TN, Hixson JE, et al. Genetic variants in the renin-angiotensin-aldosterone system and salt sensitivity of blood pressure. J Hypertens 2010; 28: 1210–1220.

[7] Svetkey LP, Harris EL, Martin E, et al. Modulation of the BP response to diet by genes in the reninangiotensin system and the adrenergic nervous system. Am J Hypertens 2011; 24:209–217.

[8] Citterio L, Simonini M, Zagato L, et al. Genes involved in vasoconstriction and vasodilation system affect salt-sensitive hypertension. PLoS One 2011; 6:e19620.

[9] Liu Z , Qi H , Liu B , et al. Genetic susceptibility to salt-sensitive hypertension in a Han Chinese population: a validation study of candidate genes[J]. Hypertension Research, 2017.40(10):876-884

[10] Liu K, Liu Z, Qi H, et al. Genetic Variation in SLC8A1 Gene Involved in Blood Pressure Responses to Acute Salt Loading.[J]. American Journal of Hypertension, 2018 Mar 10;31(4):415-421.

[11] Manunta P, Lavery G, Lanzani C, et al. Physiological interaction between alpha-adducin and WNK1- NEDD4L pathways on sodium-related blood pressure regulation. Hypertension 2008; 52:366–372.

[12] Liu F, Zheng S, Mu J, et al. Common variation in with no-lysine kinase 1 (WNK1) and blood pressure responses to dietary sodium or potassium interventions- family-based association study[J]. Circulation Journal, 2012, 77(1):169-174.

[13] Dahlberg J, Sjögren M, Hedblad B, et al. Genetic variation in NEDD4L, an epithelial sodium channel regulator, is associated with cardiovascular disease and cardiovascular death.[J]. Journal of Hypertension, 2014, 32(2):294-299.

[14] Barlassina C, Dal Fiume C, Lanzani C, et al. Common genetic variants and haplotypes in renal CLCNKA gene are associated to salt-sensitive hypertension. Hum Mol Genet 2007; 16:1630–1638.

[15] Fava C, Danese E, Montagnana M, et al. Serine/threonine kinase 39 is a candidate gene for primary hypertension especially in women: results from two cohort studies in Swedes. J Hypertens 2011; 29:484–491.

[16] Rhee M Y , Yang S J , Oh S W , et al. Novel genetic variations associated with salt sensitivity in the Korean population[J]. Hypertension Research, 2011, 34(5):606-611.

[17] Zhao Q, Hixson JE, Rao DC, et al. Genetic variants in the apelin system and blood pressure responses to dietary sodium interventions: a family-based association study. J Hypertens 2010; 28:756–763.

[18] Kingah P L, Luu H N, Volcik K A, et al. Association of NOS3 Glu298Asp SNP with hypertension and possible effect modification of dietary fat intake in the ARIC study[J]. Hypertension Research Official Journal of the Japanese Society of Hypertension, 2010, 33(2):165-169.

[19] Caprioli J, Mele C, Mossali C, et al. Polymorphisms of EDNRB, ATG, and ACE genes in salt-sensitive hypertension. Can J Physiol Pharmacol 2008; 86:505–510.

[20] Defagó MD, Gu D, Hixson JE,et al. Common genetic variants in the endothelial system predict blood pressure response to sodium intake: the GenSalt study. Am J Hypertens. 2013 May;26(5):643-656.

[21] Castejon AM, Bracero J, Hoffmann IS, et al. NAD(P)H oxidase p22phox gene C242T polymorphism, nitric oxide production, salt sensitivity and cardiovascular risk factors in Hispanics. J Hum Hypertens 2006; 20:772–779.

[22] Armando I, Villar V A, Jose P A. Genomics and Pharmacogenomics of Salt-sensitive Hypertension[J]. Current Hypertension Reviews, 2015;11(1):49-56.

[23] Kelly TN, Rebholz CM, Gu D,et al.Analysis of sex hormone genes reveals gender differences in the genetic etiology of blood pressure salt sensitivity: the GenSalt study. Am J Hypertens. 2013 Feb;26(2):191-200.

[24] Kelly TN, Rice TK, Gu D, et al. Novel genetic variants in the alpha-adducin and guanine nucleotide binding protein beta-polypeptide 3 genes and salt sensitivity of blood pressure. Am J Hypertens 2009; 22:985–992.

[25] Citterio L, Ferrandi M, Delli C S, et al. cGMP-dependent protein kinase 1 polymorphisms underlie renal sodium handling impairment.[J]. Hypertension, 2013, 62(6):1027-1033.

[26] Pojoga L, Kolatkar N S, Williams J S, et al. Beta-2 adrenergic receptor diplotype defines a subset of salt-sensitive hypertension.[J]. Hypertension, 2006, 48(5):892.

[27] Sun B, Williams J S, Svetkey L P, et al. β2-Adrenergic receptor genotype affects the renin-angiotensin-aldosterone system response to the Dietary Approaches to Stop Hypertension (DASH) dietary pattern[J]. American Journal of Clinical Nutrition, 2010, 92(2):444-449.

[28] Sanada H, Yatabe J, Midorikawa S, et al. Single-nucleotide polymorphisms for diagnosis of saltsensitive hypertension. Clin Chem 2006; 52:352–360.

[29] Mei H, Gu D, Hixson JE,et al. Genome-wide linkage and positional association study of blood pressure response to dietary sodium intervention: the GenSalt Study. Am J Epidemiol. 2012 Oct 1;176 Suppl 7:S81-90.

[30] He J, Kelly TN, Zhao Q,et al.Genome-wide association study identifies 8 novel loci associated with blood pressure responses to interventions in Han Chinese. Circ Cardiovasc Genet. 2013 Dec;6(6):598-607.

[31] Li C, Yang X, He J,et al. A gene-based analysis of variants in the serum/glucocorticoid regulated kinase (SGK) genes with blood pressure responses to sodium intake: the GenSalt Study. PLoS One. 2014 May 30;9(5):e98432.

[32] Warnock D G, Kuschevihrog K, Tarjus A, et al. Blood pressure and amiloride-sensitive sodium channels in vascular and renal cells.[J]. Nature Reviews Nephrology, 2014, 10(3):146-157.

[33] Eap CB, Bochud M, Elston RC, et al. CYP3A5 and ABCB1 genes influence blood pressure and response to treatment, and their effect is modified by salt. Hypertension 2007; 49:1007–1014.

Table S2 Effect size of SNPs associated with salt sensitivity

| gene | SNP | |  | |  | | SR n(%) | SS n(%) | *P* ^¤^ | | *P* ^§^ | OR | | 95%CI | |
| --- | --- | --- | --- | --- | --- | --- | --- | --- | --- | --- | --- | --- | --- | --- | --- |
| STK39 | | rs3754777 | | genotype | | TT | 45(8.5) | 9(4.3) |  | ref | | | ref | ref |  |
|  | |  | |  | | CT | 209(39.4) | 72(34.1) |  | 0.145 | | | 1.773 | 0.820-3.833 |  |
|  | |  | |  | | CC | 277(52.2) | 130(61.6) | 0.027* | 0.020* | | | 2.433 | 1.148-5.153 |  |
|  | |  | | additive | |  |  |  | 0.050* | 0.042* | | | 1.467 | 1.013-2.124 |  |
|  | |  | | recessive | | CT+CC vs. TT | 486(91.5)/45(8.5) | 202(95.7)/9(4.3) | 0.046* | 0.042* | | | 2.155 | 1.028-4.516 |  |
|  | |  | | dominant | | CC vs. TT+CT | 277(52.2)/254(47.8) | 130(61.6)/81(38.4) | 0.020* | 0.017* | | | 1.489 | 1.074-2.066 |  |
|  | |  | |  | |  |  |  |  |  | | |  |  |  |
| KLK1 | | rs5516 | | genotype | | GG | 332(62.8) | 118(56.2) |  | ref | | | ref | ref |  |
|  | |  | |  | | CG | 177(33.4) | 77(36.7) |  | 0.253 | | | 1.220 | 0.867-1.717 |  |
|  | |  | |  | | CC | 20(3.8) | 15(7.1) | 0.078 | 0.041* | | | 2.091 | 1.030-4.245 |  |
|  | |  | | additive | |  |  |  | 0.036* | 0.052 | | | 1.307 | 0.997-1.712 |  |
|  | |  | | recessive | | CC vs. CG+GG | 20(3.8)/509(96.2) | 15(7.1)/195(92.9) | 0.052 | 0.061 | | | 0.514 | 0.256-1.032 |  |
|  | |  | | dominant | | CC+CG vs. GG | 197(37.2)/332(62.8) | 92(43.8)/118(56.2) | 0.099 | 0.105 | | | 0.764 | 0.551-1.058 |  |
|  | |  | |  | |  |  |  |  |  | | |  |  |  |
| SLC24A3 | | rs6112470 | | genotype | | CC | 22(4.2) | 8(3.8) |  | ref | | | ref | ref |  |
|  | |  | |  | | CT | 207(39.0) | 57(27.0) |  | 0.492 | | | 0.738 | 0.311-1.753 |  |
|  | |  | |  | | TT | 301(56.8) | 146(69.2) | 0.007* | 0.535 | | | 1.303 | 0.564-3.010 |  |
|  | |  | | additive | |  |  |  | 0.006* | 0.007* | | | 1.507 | 1.118-2.031 |  |
|  | |  | | recessive | | CT+TT vs. CC | 508(95.8)/22(4.2) | 203(96.2)/8(3.8) | 0.823 | 0.865 | | | 1.075 | 0.469-2.464 |  |
|  | |  | | dominant | | TT vs. CC+CT | 301(56.8)/229(43.2) | 146(69.2)/65(30.8) | 0.002* | 0.020* | | | 1.718 | 1.223-2.415 |  |
|  | |  | |  | |  |  |  |  |  | | |  |  |  |
| WNK1 | | rs12828016 | | genotype | | GG | 41(7.6) | 14(6.7) |  | ref | | | ref | ref |  |
|  | |  | |  | | GT | 214(39.9) | 66(31.6) |  | 0.746 | | | 0.895 | 0.458-1.750 |  |
|  | |  | |  | | TT | 282(52.5) | 129(61.7) | 0.073 | 0.347 | | | 1.363 | 0.715-2.597 |  |
|  | |  | | additive | |  |  |  | 0.049* | 0.038* | | | 1.325 | 1.016-1.727 |  |
|  | |  | | recessive | | TT vs. GT+GG | 41(7.6)/496(92.4) | 14(6.7)/195(93.3) | 0.756 | 0.645 | | | 1.160 | 0.617-2.183 |  |
|  | |  | | dominant | | GG vs. GT+TT | 282(52.5)/255(47.5) | 129(61.7)/80(38.3) | 0.027* | 0.017 | | | 1.494 | 1.076-2.076 |  |
|  | |  | |  | |  |  |  |  |  | | |  |  |  |
| VSNL1 | | rs16983422 | | genotype | | AA | 417(77.9) | 148(70.5) |  | ref | | | ref | ref |  |
|  | |  | |  | | GA | 106(19.9) | 56(26.6) |  | 0.042* | | | 1.478 | 1.015-2.153 |  |
|  | |  | |  | | GG | 12(2.2) | 6(2.9) | 0.100 | 0.770 | | | 1.172 | 0.404-3.399 |  |
|  | |  | | additive | |  |  |  | 0.047* | 0.076 | | | 1.331 | 0.971-1.825 |  |
|  | |  | | recessive | | GG vs. GA+AA | 12(2.2)/523(97.8) | 6(2.9)/204(97.1) | 0.623 | 0.654 | | | 0.796 | 0.293-2.162 |  |
|  | |  | | dominant | | GG+GA vs. AA | 118(22.1)/417(77.9) | 62(29.5)/148(70.5) | 0.032* | 0.036* | | | 1.473 | 1.025-2.115 |  |
|  | |  | |  | |  |  |  |  |  | | |  |  |  |
| AGTR1 | | rs2638360 | | genotype | | AA | 433(81.2) | 155(74.5) |  | ref | | | ref | ref |  |
|  | |  | |  | | GA | 94(17.7) | 47(22.6) |  | 0.113 | | | 1.380 | 0.927-2.055 |  |
|  | |  | |  | | GG | 6(1.1) | 6(2.9) | 0.059 | 0.067 | | | 2.938 | 0.927-9.309 |  |
|  | |  | | additive | |  |  |  | 0.021* | 0.019* | | | 1.497 | 1.068-2.100 |  |
|  | |  | | recessive | | GG vs. GA+AA | 6(1.1)/527(98.9) | 6(2.9)/202(97.1) | 0.088 | 0.084 | | | 0.362 | 0.115-1.145 |  |
|  | |  | | dominant | | GG+GA vs. AA | 100(18.8)/433(81.2) | 53(25.5)/155(74.5) | 0.042* | 0.048* | | | 1.471 | 1.004-2.157 |  |

Abbreviations: SNP, Single nucleotide polymorphisms; SS, Salt sensitive; SR, Salt resistant.

^§^: Chi-square test was used.

^¤^: Logistic regression was used. Adjusted for age, sex and hypertension.

**P*≤0.05
